# Association of angiotensinogen gene SNPs and haplotypes with risk of hypertension in eastern Indian population

**DOI:** 10.1186/s40885-017-0069-x

**Published:** 2017-03-29

**Authors:** Pulakes Purkait, Kalpataru Halder, Sunil Thakur, Abhishikta Ghosh Roy, Pradip Raychaudhuri, Sandip Bhattacharya, B. N. Sarkar, J. M. Naidu

**Affiliations:** 1 DNA Laboratory, Anthropological Survey of India, Western Regional Center, Udaipur, 313001 Rajasthan India; 2 Department of Molecular Biology, BrahmanandaKeshab Chandra College, 111/2 B.T.Road, BonHooghly, P.O. - BonHooghly, Kolkata, 700 108 West Bengal India; 3 Department of Anthropology, University of Delhi, North Campus, Delhi, 110007 India; 4 DNA Laboratory, Anthropological Survey of India, 27 Jawaharlal Nehru Road, Kolkata, 700016 India; 5 Department of Endocrinology, Calcutta Medical College & Hospital, 88, College Street, Kolkata, 700073 India; 6 Department of Nephrology & Dialysis, B.P. Poddar Hospital & Medical Research LTD, 71/1 HumayunKabirSarani, New Alipore, Block - G, Kolkata, 700053 West Bengal India; 7 Department of Anthropology, Andhra University, Visakhapatnam, 530003 Andhra Pradesh India

**Keywords:** Angiotensinogen, SNP, Haplotype, Hypertension, Methylation, Indian population

## Abstract

**Background:**

Angiotensinogen (AGT) enzyme comprises a vital module of RAAS system that effectively controls the blood pressure and related cardiovascular functions. Ample association studies have reported the importance of AGT variants in cardiovascular and non-cardiovascular adversities. But lately, owing to the complexity of the many anomalies, the haplotype based examination of genetic variation that facilitates the identification of polymorphic sites which are located in the vicinity of the causative polymorphic site, gets greater appreciation.

**Methods:**

In the present study, we have done genotype and haplotype analysis of AGT gene in reference to hypertension to confirm the association of the two in an Indian population. To accomplish this, we performed candidate SNPs analysis and construct possible haplotypes across the *AGT* promoter and gene region in 414 subjects (256 Hypertensive cases and 158 controls).

**Results:**

We found four SNPs (rs11568020: A-152G and rs5050: A-20C in promoter; rs4762 and rs699 in exon2) and 3 haplotypes (H4, H7 and H8) that showed a stronger positive association with hypertension. The haplotype H2 was showing protective association with hypertension.

**Conclusion:**

The results of the present study confirmed and reestablished the role of AGT gene variants and their haplotypes in the causation of hypertension in Indian population and showed that haplotypes can provide stronger evidence of association.

**Electronic supplementary material:**

The online version of this article (doi:10.1186/s40885-017-0069-x) contains supplementary material, which is available to authorized users.

## Background

Hypertension (HTN) is a chronic medical condition in which the blood pressure in the arteries is increased. It is one of the most common and complex human diseases that cause significant heart failure, renal failure, ventricular arrhythmias, blindness and other serious medical problems [1, 2]. It is a common risk factor for cardiovascular morbidities like stroke, atherosclerosis and myocardial infarction. In a worldwide analysis of the global problem of HTN, 20.6% of Indian men and 20.9% of Indian women were suffering from HTN in 2005 [3, 4]. This problem of HTN is going to worsen in the near future as the rates for HTN are projected to go up to 22.9 and 23.6% in Indian men and women by 2025 [4].

As the blood pressure is regulated by the surrounding environment and also by the genetics of individuals, the genetic basis of primary hypertension became complex due to the interaction of the two. For a complex trait the susceptible gene(s) are searched by genetic-association studies. These studies look for deviations from the random occurrence of the alleles with respect to disease phenotype which consequently results in significant increase or decrease in their frequency. Allelic association can explain either by direct biological action of the allele (SNP analysis) or by linkage disequilibrium (LD) with a nearby susceptibility gene (haplotype analysis). SNP analysis is pretty widely used for the disorder with single gene origin. However the complex nature of disorder renders the exact genetic cause in assumptive association and increases the complexity of understanding. For such complex traits, recently the LD based examination of genetic variation (haplotype analysis) that facilitates the identification of polymorphic sites which are located in the vicinity of the causative polymorphic site, gets greater appreciation [1, 5]. LD occurs when a particular marker allele lies so close to the disease-susceptibility allele that these alleles will be inherited together over many generations [6].

Among candidate genes for primary hypertension, AGT was the first and one of the most examined genes associated with it [7]. The human AGT gene is a member of the serpin gene superfamily, which extends only 12 kb with 5 exons on chromosome 1 (1q42-q43). It is equally diverse in its cell specificity as it expressed in multiple tissues, including the liver, adipose tissue, heart, vessel wall, brain, and kidney [8]. Functionally, AGT act as a substrate to rennin enzyme, a part of Renin-Angiotensin-Aldosterone System (RAAS), where N-terminal amino acids of mature AGT secreted by hepatocytes are cleaved intravascular. First cleaved by renin, released from juxtaglomerular cells, to yield the angiotensin-I decapeptide, and then by angiotensin converting enzyme (ACE) to generate the angiotensin II (Ang II) octapeptide. The renin–AGT enzymatic reaction is the rate-limiting step of the RAAS cascade which controls the plasma AGT levels and crucial for maintaining blood pressure [7].

The most convincing early genetic evidence implicating this gene in essential hypertension in humans has revealed a number of polymorphisms in the 5′ flanking region, exons, and introns of the gene [7]. Zhao et al., also showed the functional implication of nucleotides −20, −17, −517, and −792 of AGT in the pathogenesis of high blood pressure [9]. Several other studies pointed out a correlation of plasma AGT levels, anti-AGT antibodies, injection of AGT and AGT transgenes with blood pressure [10–13]. All these studies laid foundation and make AGT gene a perfect marker to study its haplotype association with the pathogenesis of hypertension. In this context, a study identified 44 SNPs in the AGT gene and assembled a complete haplotype map with six major haplotypes of *AGT* from whites and Japanese which accounts for most of the variation in the AGT gene, although the frequency of each differed substantially in the two populations [14]. Further, Zhu et al., also constructed a haplotype map of each gene of the RAAS in black and white hypertensive populations [15, 16]. He further performed association analysis with individual SNPs and haplotype blocks and found a positive association with several SNPs in *AGT* gene with hypertension, though there was no transmission distortion of any particular haplotype for AGT [15]. Contrasting results were obtained in another report evaluating the association between haplotype blocks of AGT and their interaction with the ACE locus in a Taiwanese population [6]. These studies, however, hint toward the association of haplotypes with the hypertension, but leaving the complexity of this association difficult to interpret.

In the present study, we wish to provide further resolution of the contribution of *AGT* genetic variation (both SNPs and haplotype) to hypertension in the context of Indian population. Based on the foundation laid by earlier studies, the present study hypothesized that the AGT genotype and haplotype will also associate with hypertension in Indian population. Further, to the best of our knowledge this is the first report of association of individual SNPs and haplotype blocks of *AGT* with hypertension in an Eastern Indian population.

## Methods

### Study patients

The present study is a cross-sectional case control study consisted of 256 hypertensive patients and 158 controls from the ethnic Bengali speaking population of Kolkata city and surrounding area, West Bengal in Eastern India. Registered patients were recruited from two participating medical institutions, namely Calcutta Medical College and Hospital (Kolkata, West Bengal) and B.P. Poddar Hospital and Research Centre (Kolkata, West Bengal). Ethical committee clearance was obtained from the respective medical institutions and Ethical committee of the Anthropological Survey of India, Govt. of India. Prior to the recruitment of subjects and sample collection, an informed written consent was obtained from all the participants. The identification of hypertensive patients was based on the physician’s recommendation or registered patient for antihypertensive drugs. Blood samples of the control group were obtained from non-hypertensive individuals that were randomly selected based on physical examinations during May to September 2010. For the controls, selection criteria included; no individual history of high blood pressure, gender matched to cases and individuals were unrelated.

### Anthropometric, physiological and biochemical data

Both, patients and controls were anthropometrically measured for height vertex (cm), and weight (kg) using standard methodology [17, 18]. Body mass index (BMI) was calculated using the formula, weight (kg)/[height (m^2^)]. Clinical information regarding duration of diabetes, presence of any complication and history of other disorders was recorded. Systolic blood pressure (SBP) and diastolic blood pressure (DBP) were measured on the right arm of the subjects in sitting position using an automated blood pressure monitor (Omron, Japan) after 15 min of rest.

Approximately 10 ml of peripheral venous blood sample was collected from each individual participated in the study into two separate tubes, one in a 6 ml BD K2 vacutainer® (BD, NJ, USA) containing EDTA as an anticoagulant for genetic analysis and another in a 4 ml BD Serum vacutainer® without EDTA for biochemical analysis. Blood samples were stored at 4 °C to avoid haemolysis and cellular damage. Samples were transported to the laboratory within 3 h of collection to ensure good results. Thereafter, blood samples were transferred to labeled sterile polypropylene centrifuge tubes. The blood samples were centrifuged and serum was separated and stored at 4 °C as well as at −86 °C until further analysis. Blood glucose was measured using the *Breez 2 glucometer* (blood glucose monitor) in the field itself. All laboratory tests were conducted at the DNA laboratory in the Anthropological Survey of India. The levels of total cholesterol, triglycerides, high-density lipoprotein cholesterol (HDL), low density lipoprotein cholesterol (LDL), urea, uric acid, creatinine, chloride, total protein and albumin in serum were measured enzymatically on an auto analyzer EM360 (TRANSASIA) with the help of kits supplied by Transasia Bio-Medical LTD for the purpose. Genomic DNA was prepared from fresh whole blood sample collected in EDTA containing tube by using the conventional phenol-chloroform extraction method followed by ethanol precipitation [19] and the DNA quantity and quality was checked by both spectrophotomery and agarose gel electrophoresis. The DNA samples were stored at −20 °C to −80 °C as per the period of usage.

### Genotyping

**Fig. 1:**
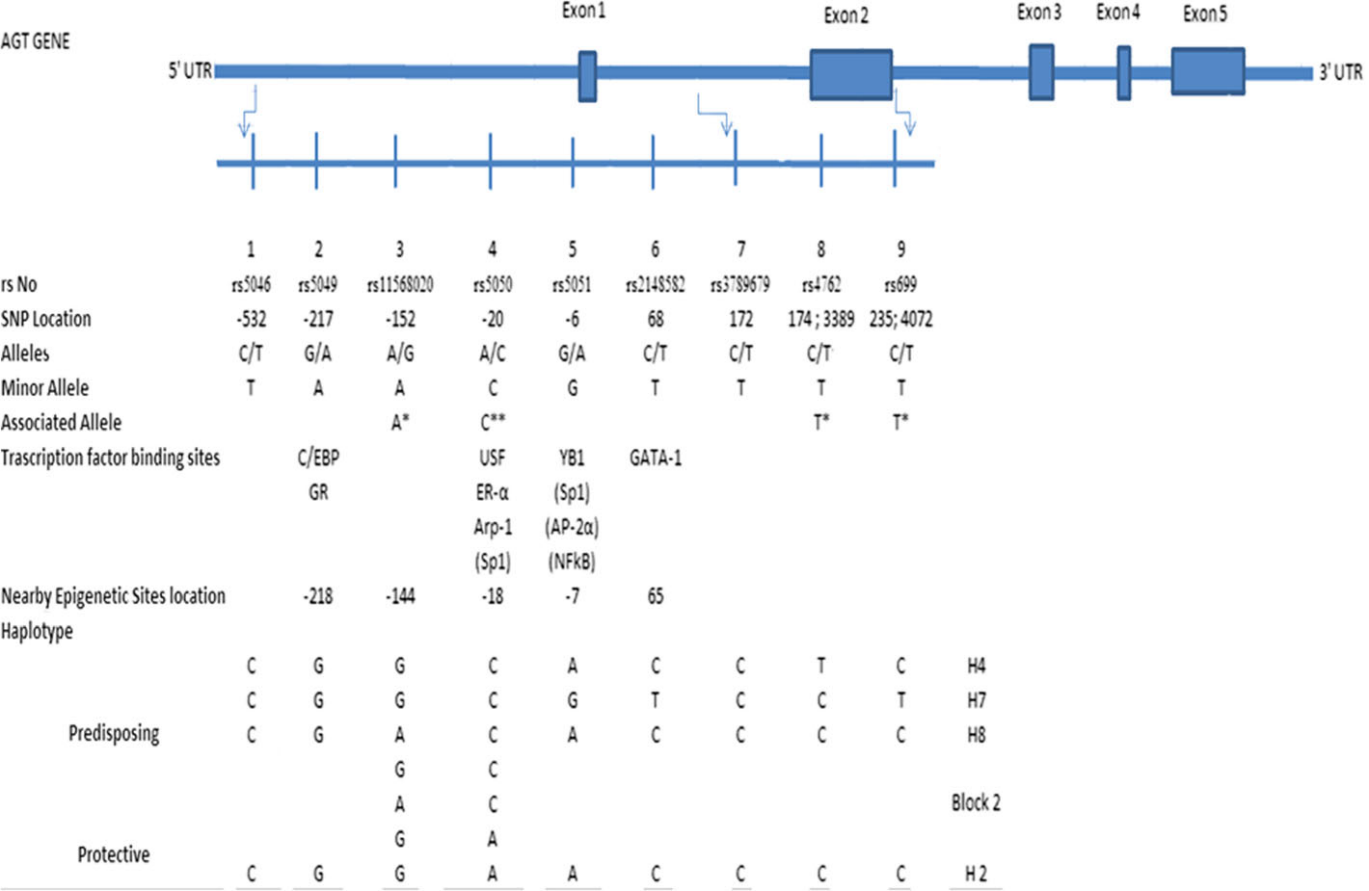
Schematic map of angiotensinogen (AGT) gene. Structure of the human AGT gene with common SNPs depicted from the promoter, Intron and Exon 2 regions. Known transcription factor binding sites and Methylation site overlying SNPs are shown below the corresponding SNP. Putative transcription factor binding sites and Haplotypes are in parentheses. UTR indicates untranslated region [1, 5, 28, 29, 38]

For the present study, our region of interest was the 5’ UTR, exon, and intron region of AGT gene. Therefore, the study included Nine SNPs (rs5046 (5'UTR), rs5049 (5'UTR), rs11568020 (5'UTR), rs5050 (5'UTR), rs5051 (5'UTR), rs2148582 (intron), rs3789679 (intron), rs4762 (exon 2), rs699 (exon 2), searched through the *Ensemble genome browser,* NCBI SNPs database and the HapMap database (Fig. 1). In this present study, except self designed AGT PRO primer, previously published primers were used for the PCR based detections of SNPs [14, 20].

Only those PCR products that had a single amplification product with no evidence of non-specific amplification were used for DNA sequencing. The samples were analyzed on ABI 3730 genetic analyzer with a 48 capillary (Applied Biosystems, USA) to generate DNA sequences. After completion of sequencing reaction, the generated sequences were checked by using *Sequencing Analysis v5.2 software* (Applied Biosystems, USA), and sequences were aligned to their respective reference sequences with the use of *SeqScape v2.5 software* (Applied Biosystems, USA), for automated sequence data analysis. It performs sequence comparisons for variant identifications, SNP discovery and validation. Haplotype construction was done using Haploview 4.2 software [21]. A total of 13 possible haplotypes (H1 to H13) was constructed for association analysis.

### Statistical analysis

Descriptive statistics were calculated for all the anthropometric and clinical variables. Mean differences between case and control groups for the continuous variables were calculated using t- test. A level of *p* < 0.05 was assumed as statistically significant. All the above analyses were performed using SPSS v.16 software (SPSS Inc., Chicago, IL, USA). Allele frequencies were calculated for the SNPs and tested for Hardy-Weinberg equilibrium (HWE) and allelic association with disease (Fisher exact test) using PLINK software package [22]. Genotype association with the phenotypes was tested under different genetic models by regression analysis. For comparing the allelic distributions between study groups, the odds ratio (OR) with 95% confidence interval (CI) were also calculated. Haploview 4.2 software was used to evaluate LD and construct haplotypes [21]. Haplotypes were constructed from genotype data in the full-size case–control panel within blocks to find their association with hypertension risk using an EM algorithm method with Haploview 4.2 software. LD between the nine SNPs used in haplotype analysis was measured by a pairwise D' statistic. The structure of the LD block (with 80% CI) was examined using the solid spine and custom methods in LD analysis Haploview 4.2 software [21, 23].

## Results

### Subject’s characteristics

As per the disease, the descriptive statistics of metric variables were shown in Table 1. Comparatively, the mean age, blood pressure, LDL, Uric acid, Urea, BUN, chloride, glucose and cholesterol in Hypertensive group were significantly higher than Normotensive group. On the other hand, higher values of BMI, triglyceride and HDL have also been found among the Hypertensives but statistically non-significant.

**Table 1:** Clinical characteristics of the study groups

Variables	Normotensive (*n* = 158)		Hypertensive (*n* = 256)		*t*-test (*p*-value)
	Mean	SE	Mean	SE	
Age (YEAR)	53.03	0.41	56.45	0.51	*0.000*
Body Mass Index (BMI) (Kg/m^2^)	23.45	0.31	24.22	0.28	0.075
SBP (mm of mercury)	105.37	0.61	161.38	1.08	*0.000*
DBP (mm of mercury)	75.51	0.67	91.98	0.74	*0.000*
Glucose(mg/dl)	120.77	3.71	132.50	3.70	*0.035*
Cholesterol (mg/dl)	167.19	2.75	177.82	2.70	*0.009*
Triglycerides (mg/dl)	153.38	5.81	162.83	5.08	0.233
HDL (mg/dl)	45.73	1.40	47.71	1.07	0.259
LDL (mg/dl)	90.79	1.94	97.66	2.03	*0.022*
Uric Acid (mg/dl)	5.48	0.11	6.10	0.10	*0.000*
Urea(mg/dl)	22.40	1.25	46.44	2.28	*0.000*
BUN (mg/dl)	10.46	0.58	21.69	1.06	*0.000*
Chloride (mmol/L)	103.92	0.76	110.10	0.84	*0.000*

Significance values are italicized, Level of significance < 0.05

### Association analyses of AGT polymorphism and hypertension

All the SNPs were polymorphic with minor allele frequencies > 5% and genotype distributions in agreement with Hardy-Weinberg equilibrium among the Normotensive control group (Table 2). Two of the SNPs rs699 and rs4762 were non-synonymous mutations, the rest SNPs were in the untranslated regions or intron of the gene (Additional file 1: Table S1). The association analysis of hypertension (256 cases versus 158 controls) demonstrated the causative associations of rs11568020 A [Odds ratio (OR) = 6.382; *p*-value (p) = 0.003], the rs5050 C [OR = 2.808; *p* = 0.000] and the rs4762 T [OR =1.57; *p* = 0.034] variants with the disease (Table 3).

**Table 2:** Genotype distribution and HWE tests for all nine diallelic polymorphisms in the AGT Gene

dbSNP ID	Minor Allele (A1)	A2	Group	Genotype Distribution		HWE		
				GENO	*p*-value	O(HET)	E(HET)	*p*-value
rs5046	T	C	Hypertensive	14/90/152	0.655	0.3516	0.3547	0.861
			Normotensive	12/52/94		0.3291	0.3653	0.198
rs5049	A	G	Hypertensive	14/94/148	0.684	0.3672	0.363	1
			Normotensive	12/56/90		0.3544	0.3781	0.406
rs11568020	A	G	Hypertensive	0/20/236	*0.004*	0.07812	0.07507	1
			Normotensive	0/2/156		0.01266	0.01258	1
rs5050	C	A	Hypertensive	34/74/148	*0.000*	0.2891	0.4008	*0.000*
			Normotensive	4/30/124		0.1899	0.2116	0.244
rs5051	G	A	Hypertensive	28/90/138	0.292	0.3516	0.4077	*0.031*
			Normotensive	12/66/80		0.4177	0.4074	0.846
rs2148582	T	C	Hypertensive	26/90/140	0.170	0.3516	0.4008	0.060
			Normotensive	10/68/80		0.4304	0.4019	0.433
rs3789679	T	C	Hypertensive	0/50/206	*	0.1953	0.1762	0.145
			Normotensive	2/24/132		0.1519	0.1615	0.342
rs4762	T	C	Hypertensive	8/70/178	*0.000*	0.2734	0.2795	0.659
			Normotensive	0/36/122		0.2278	0.2019	0.224
rs699	T	C	Hypertensive	28/88/140	*0.023*	0.3438	0.4043	0.019
			Normotensive	8/72/78		0.4557	0.4019	0.114

Significance values are italicized, Level of significance <0.05; Chi-Sq = 4.384; Degrees of freedom (DF) = 2; 1 cells with expected counts less than 1.0;*Chi-Square approximation probably invalid; 2 cells with expected counts less than 5.0

**Table 3:** Fisher exact test for the study group Normotensive and Hypertensive

SNP	Minor allele	Frequency		Odd Ratio (95% CI)	*p*-value
		Hypertensive	Normotensive		
rs5046	T	0.2305	0.2405	0.9458 (0.68 - 1.315)	0.736
rs5049	A	0.2383	0.2532	0.9228 (0.6668-1.277)	0.677
rs11568020	A	0.03906	0.006329	6.382 (1.482-27.49)	*0.003*
rs5050	C	0.2773	0.1203	2.808 (1.9-4.148)	*0.000*
rs5051	G	0.2852	0.2848	1.002 (0.7342-1.367)	1
rs2148582	T	0.2773	0.2785	0.9943 (0.7272-1.36)	1
rs3789679	T	0.09766	0.08861	1.113 (0.6851-1.809)	0.714
rs4762	T	0.168	0.1139	1.57 (1.034-2.383)	*0.034*
rs699	T	0.2812	0.2785	1.014 (0.7418-1.386)	1

Significance values are italicized, Level of significance < 0.05

The associations were further verified via regression analysis through 3 genotypic model tests; additive model (ADD), dominant model (DOM) and recessive model (REC) to confirm the predictive association between both study groups. ADD and DOM models showed significant association with hypertension for the SNP rs11568020 (ADD: OR = 6.61, *p* = 0.012; DOM: OR = 6.61, *P* =0.012) and rs4762 (ADD: OR = 1.587, *p* = 0.032), while the recessive model shows significant association and risk with the hypertension for the SNP rs699 (OR = 2.303; *p* = 0.04417). The SNP rs5050 is showing association with hypertension in all three genotypic models (ADD: OR = 2.339, *p* = 0.000; DOM: OR = 2.661, *p* = 0.000 and REC: OR = 5.896; *p* = 0.001) (Table 4).

**Table 4:** Logistic regression analysis between Normotensive and Hypertensive group

Test	SNP	A1	OR	P
Additive model	rs5046	T	0.948	0.745
	rs5049	A	0.9241	0.631
	rs11568020	A	*6.61*	*0.011*
	rs5050	C	*2.339*	*0.000*
	rs5051	G	1.002	0.991
	rs2148582	T	0.9946	0.972
	rs3789679	T	1.119	0.657
	rs4762	T	*1.587*	*0.032*
	rs699	T	1.013	0.932
Dominant model	rs5046	T	1.005	0.981
	rs5049	A	0.9658	0.865
	rs11568020	A	*6.61*	*0.011*
	rs5050	C	*2.661*	*0.000*
	rs5051	G	0.877	0.517
	rs2148582	T	0.8498	0.422
	rs3789679	T	1.232	0.432
	rs4762	T	*1.485*	*0.090*
	rs699	T	0.8079	0.292
Recessive model	rs5046	T	0.7039	0.388
	rs5049	A	0.7039	0.388
	rs11568020	A	NA	NA
	rs5050	C	*5.896*	*0.001*
	rs5051	G	1.494	0.265
	rs2148582	T	1.673	0.183
	rs3789679	T	0.000	0.999
	rs4762	T	0.000	0.998
	rs699	T	*2.303*	*0.044*

Significance values are italicized, Level of significance < 0.05

### Association analyses of AGT gene haplotypes and hypertension

Haplotype analysis involves different combinations of the nine studied SNPs, employing the most prevalent 9-mer sequence H1: CGGAGTCCT (frequency = 0.211; Case = 0.201; Control = 0.229; *p* = 0.330) as reference for comparative analysis of their relationships with hypertension risk. As shown in Table 5, the haplotypes H4: CGG*C*ACCTC (*χ*2 = 7.234; *p* = 0.007), H7: CGG*C*GTCCT (*χ*2 = 11.887; *p* = 0.001) as well as haplotype H8: CG*AC*ACCCC (*χ*2 = 5.557; *p* = 0.018) constructed from all the nine SNPs were significantly associated with HTN and exhibit the causative risk for the disease. Besides this, haplotype H2: CG*GA*ACCCC (*χ*2 = 7.718; *p* = 0.005) was associated with protective effect and showed protection against HTN.

**Table 5:** Haplotype association (Custom)

Haplotype		db SNP ID									Frequencies		*p* value
		rs5046	rs5049	rs11568020	rs5050	rs5051	rs2148582	rs3789679	rs4762	rs699	Hypertensive	Normotensive	
H1	CGGAGTCCT	C	G	G	A	G	T	C	C	T	0.201	0.229	0.330
H2	CGGAACCCC	C	G	G	A	A	C	C	C	C	0.153	0.230	*0.005*
H3	TAGAACCCC	T	A	G	A	A	C	C	C	C	0.198	0.225	0.352
H4	CGGCACCTC	C	G	G	C	A	C	C	T	C	0.143	0.081	*0.007*
H5	CGGAACTCC	C	G	G	A	A	C	T	C	C	0.088	0.079	0.667
H6	CGGCACCCC	C	G	G	C	A	C	C	C	C	0.021	0.011	0.269
H7	CGGCGTCCT	C	G	G	C	G	T	C	C	T	0.047	0.004	*0.001*
H8	CGACACCCC	C	G	A	C	A	C	C	C	C	0.031	0.006	*0.018*
H9	CGGAGTCCC	C	G	G	A	G	T	C	C	C	0.015	0.026	0.297
H10	CAGAACCCC	C	A	G	A	A	C	C	C	C	0.012	0.013	0.904
H11	CGGAACCTC	C	G	G	A	A	C	C	T	C	0.017	0.027	0.349
H12	TAGCACCCC	T	A	G	C	A	C	C	C	C	0.016	0.002	0.065
H13	CGGAACCCT	C	G	G	A	A	C	C	C	T	0.019	0.026	0.545

Significance values are italicized, Level of significance < 0.05

**Fig. 2:**
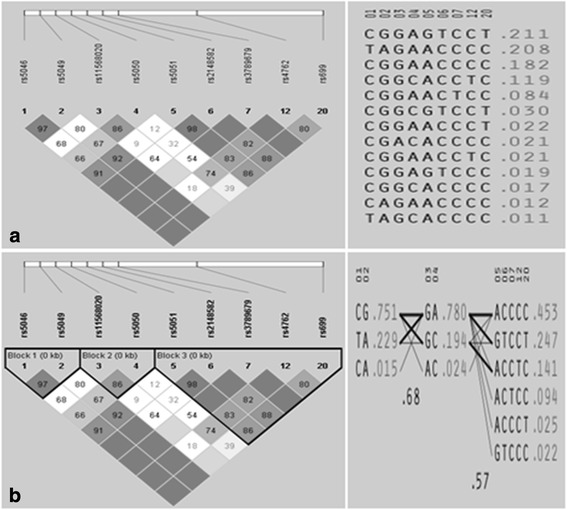
Linkage disequilibrium structure of the nine studied angiotensinogen SNPs. **a** Custom and **b** Solid Spine generated by Haploview. The SNPs are shown sequentially as they appear on the chromosome (not to scale). The value within each *square* in the *triangle* plot represents the pairwise correlation between SNPs (measured as D' = coefficient of linkage disequilibrium) defined by the *upper left* and the *upper right sides* of the *Squares*. D' for each comparison is given as the number in the square if it is not equal to 1. The Squares without a number correspond to D' = 1. The multiallelelic D' values over multiple blocks are shown between each block. Shading represents the magnitude and significance of pairwise LD, with a *gray* to *white* color gradient reflecting higher to lower LD values. The frequency of each common haplotype within a block is to the *right* of the haplotype

Further, the associations trickled down to the shorter sequences through the solid spine analysis method where all the SNPs formed three Block (Fig. 2b). Block 1 consisted of the SNPs rs5046 and rs5049, while rs11568020 and rs5050 form Block 2. The rest of the SNPs (rs5051, rs2148582, rs3789679, rs4762, rs699) were constituted of block3 [Table 6]. The highest level of significant association exhibited by Block 2 [2-mer GC (*χ*2 = 20.804; *p* =0.000)]. Apart from the haplotype GC, a second 2-mer AC (*χ*2 = 6.683; *p* = 0.0097) also associated with risk for HT, while haplotype GA (*χ*2 = 29.741; *p* =0.000) was protective against HT. The Block 1 and Block 3 showed no association between case and control groups.

**Table 6:** Haplotype association (Solid Spine)

Block	Haplotype	Freq.	Frequencies		*p* value
			Hypertensive	Normotensive	
Block 1	CG	0.751	0.758	0.740	0.575
	TA	0.229	0.226	0.234	0.8
	CA	0.015	0.012	0.019	0.396
Block 2	GA	0.78	0.718	0.880	*0.000*
	GC	0.194	0.243	0.114	*0.000*
	AC	0.024	0.034	0.006	*0.009*
Block 3	ACCCC	0.453	0.435	0.482	0.18
	GTCCT	0.247	0.248	0.244	0.901
	ACCTC	0.141	0.159	0.112	0.061
	ACTCC	0.094	0.098	0.089	0.665
	ACCCT	0.025	0.024	0.026	0.852
	GTCCC	0.022	0.020	0.026	0.571

Significance values are italicized, Level of significance < 0.05

## Discussion

The main objective of many genetic researches is to find out genes that are responsible for the particular disease. The findings of these genes should illuminate the understanding of the disease process, so that methods for preventing and treating the disease can be developed in the best possible way. For diseases with a relatively straightforward genetic basis i.e. the single-gene disorders, the current methods of genetic detection are pretty much sufficient to find the genes involved. But the problem arises with the genetic detection of multi-genetic disorders. Many common diseases such as heart disease, stroke, diabetes, cancers or psychiatric disorders have complexity in their genetic control. They are harmonized by multiple genes in coordination with environmental factors. Hence the genetic clarity in this context appears as a hazy cloud of assumptions. Although, the genetic contributions to these complex disorders are not clear, many researchers still consider the importance of common variants and follow the Common-Disease/Common-Variant theory.

Several association studies have been reported the importance of *AGT* polymorphisms and explored their association with a variety of cardiovascular and non-cardiovascular phenotypes [1, 24–26]. Hypertension is one such risk phenotype of cardiovascular disorders putatively associated with *AGT* variants [5, 27–31]. The results of the present study also found the role of *AGT* variants in susceptibility for the risk of HTN in Indian population which is similar with several other studies in other ethnic groups, including Taiwanese, Mexican, Caucasian, Chinese, Slovaks, Polish, Tunisians and Saudi [29, 32–37]. In the present study, 6.61 fold risk of rs11568020, 2.339 fold risk of rs5050, 1.587 fold risk of rs4762 (in additive and Dominant model) and 2.303 fold risk of rs699 with the occurrence of hypertension (Tables 3 and 4) suggests the role of AGT gene in hypertension. Previous studies with similar variants also showed risk association with essential hypertension [1, 5, 27, 29, 30, 38, 39]. Therefore, our study reestablishes the place of *AGT* as a risk gene for hypertension also in Indian populations.

The possible mechanistic approach which explains the results of the present study and confirm the role of two promoter SNPs, rs11568020 & rs5050 and two exon SNPs rs4762 & rs699 in the causation of hypertension gyrate around the positional significance of these associated variants in the gene. It is a well established fact that methylation levels of genes, especially methylation of CpG sites in promoter regions involve in control of gene expression, epigenetically. Mopidevi et al., showed that h*AGT* promoter CpG sites in the kidney are more methylated as compared to liver [40]. Hence the individual specific variation in tissue specific pattern might be the major cause for the occurrence of the disorders. However, the molecular mechanisms involved in tissue specific expression of this gene are not clearly understood. But the possible explanation for this phenomenon may be the DNA methylation pattern of the AGT promoter region. A number of CpG dinucleotides, the targets for DNA methylation, are located in the human *AGT* promoter. The human *AGT* promoter region has four CpG sites that correspond to the positions −218, −144, −18 and −7 (Fig. 1). The-218 CpG site of the human *AGT* promoter is the binding site of CCAAT enhancer binding protein (CEBP). Dickson et al., also showed that when the promoter at position −218/-217 is hypomethylated in tissues and cells (liver, heart and HepG2 hepatocytes) AGT is expressed in relatively higher proportion, but in hypermethylated condition (adrenal glands, leukocytes and adrenocortical H295R cells) its expression is lower [41]. Therefore, the methylation pattern of a CpG dinucleotide within the CEBP-binding site appears to be inversely associated with AGT expression. However, in the present study the SNP near to this CpG site showed no role in the causation of HTN suggesting no role of methylation at this site in the Indian populations. However, in the present study rs11568020 (at position-152) & rs5050 (at position −20) are the two nearby SNPs of the CpG sites at −144 and −18 in the promoter region. The association of these two SNPs with HTN hints toward the control of CpG methylation on their expression. The role of SNP rs5050 (A-20C) was also found to be crucial in DNA methylation on USF1/ESR1 binding to the target DNA site [41, 42, 9]. Further, this site is a binding site for most of the transcription factor of AGT gene [1, 5], hence the methylation in nearby site controls its expression and enhances the adversities associated with the AGT gene (in present context HTN). It is anticipated that the association of SNP rs11568020 (nearby the CpG site −144) with HTN may also show the similar pattern of mechanistic effect as shown by SNP rs5050 (A-20C). However, in the present study nearby sequence analysis of this position does not support the epigenetic modulation theory by DNA methylation. May be some other mechanism exist for this SNP association with essential hypertension. However to the best of our knowledge, no studies have been done regarding DNA-protein interaction in the SNP rs11568020 at position -152A/G. Future studies in Indian populations may be done to illuminate in this regard.

Haplotype analysis is a powerful tool for identifying candidate genes for complex trait disease. The haplotype analysis of the nine variants of AGT considered in the present study showed contrasting haplotype profiles of hypertensive and normotensive with significant difference between the two (Tables 5 and 6). Haplotype analyses of the AGT gene and its association with hypertension have also been reported in whites and Japanese [27, 43]. Jeunemaitre et al., have shown that ancestral T235/A-6 haplotype was associated with hypertension [27]. Sato et al., also identified 8 SNPs and demonstrated that only M235/G-6 haplotype was significantly associated with a hypotensive effect [43]. In Indian context, similar finding was reported by Patnaik et al. in 2015 [44]. The results of the present study confirmed the associations of AGT gene haplotype in Indian population as three haplotypes; haplotypes H4: CGG***C***ACCTC H7: CGG***C***GTCCT and haplotype H8: CG*A* ***C***ACCCC, were significantly associated with HTN and exhibit the causative risk for the disease (*p*-value <0.001). Haplotype H4 consist of two mutant alleles (rs5050C and rs4762T) one from promoter region and one from Exon respectively. Haplotype H7 consists of four mutant alleles (rs5050C, rs5051G, rs2148582T and rs699T) two are from the promoter and two are from exon respectively. Similar to H4 haplotype H8 haplotype is consists of two mutant alleles (rs5050C and rs11568020A), but both are from promoter region. One interesting observation about these positively associated haplotypes is that they all have a common SNPs rs5050. The involvement of rs5050 in haplotypes suggests that its role in linkage is much deeper as we saw in the individual SNPs association (Table 3). Hence the role of rs5050 (at position −20) with HTN is further confirmed even more strongly with haplotype analysis in the present study suggesting the importance of haplotype analysis method in genetic variant analysis in multi-gene anomalies. Besides this, haplotype H2: CG*GA*ACCCC consist of all wild type alleles, was significantly associated with Normotensive and showed a protective effect (*p*-value < 0.05). In the Indian context (more specifically Eastern Indians) this haplotype can be considered as ancestral haplotype of AGT.

Further, Tracking the shorter haplotypes down to the 2-mers e. g. Block 2, (Fig. 2b) indicates clearly that A allele of rs11568020 and C allele of rs5050 were consistently associated with causative effects, while its complementary rs11568020 G allele and rs5050 A allele consistently mediates protective actions. Both the SNPs are situated at the core promoter region of the AGT gene and therefore pointing the role of these loci as a central component linking AGT with Hypertension. This association might be regulated via the methylation pattern of AGT gene.

## Conclusion

The identification of associated genes in the causation of complex disorders is very difficult and even more complex process. Over the past few decades, tremendous efforts have been made to solve the complexity of disorders like hypertension. The association of SNPs and their haplotypes with hypertension in world populations showed some promising results in solving this problem to some extent. The association of AGT SNPs and haplotype with HTN in the present study confirmed the role of AGT in HTN in Indian population. Further, the central role of AGT rs5050 SNP in the causation of HTN in the present study showed even more strong evidence for the importance of haplotype analysis. The present study also pointed out the possible role of methylation pattern and its interaction with SNPs in the promoter region of AGT in the causation of hypertension. However, more haplotype based and in depth studies are needed for the identification of genetic variants in complex disorders. Future studies with defined experimental models, where the interaction between genetic and environmental risk factors will consider, might help in this context.

## Additional file

Additional file 1: Table S1. Important characteristic of the studied SNPs. (DOC 146 kb)

## Abbreviation

A1: Code for allele 1 (the more rare or ‘minor’ allele based on the entire sample frequencies)
A2: Code for allele 2 (the more common or ‘major’ allele)
ACE: Angiotensin converting enzyme
ADD: Additive model
AGT: Angiotensinogen
Ang II: Angiotensin II
BMI: Body mass index
BUN: Blood urea nitrogen
CEBP: Enhancer binding protein
CI: Confidence interval
CpG: Cytosine-guanine dinucleotides
DBP: Diastolic blood pressure
DOM: Dominant model
E(HET): Expected frequency of heterozygotes
GENO: Genotype
h*AGT*: Human AGT
HDL: High-density lipoprotein cholesterol
HTN: Hypertension
HWE: Hardy-Weinberg equilibrium
LD: Linkage disequilibrium
LDL: Low density lipoprotein cholesterol
NCBI: National Centre for Biotechnology Information
O(HET): Observed frequency of heterozygotes
OR: Odds ratio
p: The asymptotic *p*-value for chi-square test
PCR: Polymerase chain reaction
RAAS: Renin-angiotensin-aldosterone system
REC: Recessive model
rs: Reference SNP ID number
SBP: Systolic blood pressure
SNP: Single nucleotide polymorphism
UTR: Untranslated regions
