# Supplementary material for: Association of angiotensinogen gene SNPs and haplotypes with risk of hypertension in eastern Indian population
Source: Clin Hypertens. 2017 Mar 29;23:12. doi: 10.1186/s40885-017-0069-x (PMC5371191; doi:10.1186/s40885-017-0069-x)
Supplement: Supplementary file 1 [file 40885_2017_69_MOESM1_ESM.doc]

Additional file - S1: Important characteristic of the studied SNPs

	Gene Locus	db SNP ID	Variation	Nearby methylation site	AA change	Chromosomal Loci	MAF (case) Present Study	HapMap MAF		
								CEU	CBH	YRI
**1**	promoter	rs5046	(-532) C / T	na	Na	1:230850398	T (0.2305)	0.11	0.21	0.16
**2**	promoter	rs5049	(-217) G/A	-218	Na	1:230850083	A (0.2383)	0.11	0.19	0.29
**3**	promoter	rs11568020*	(-152) A/G	-144	Na	1:230850018	A (0.03906)	0.04	0.05	0.01
**4**	promoter	rs5050*	(-20) A / C	-18	Na	1:230849886	C (0.2773)	0.14	0.17	0.12
**5**	promoter	rs5051	(-6) G/A [T>C]	-7	Na	1:230849872	G (0.2852)	0.4(T)	0.28(C)	0.06(C)
**6**	Intronic	rs2148582	(+68) T/C [C>T]	+65	Na	1:230849799	T (0.2773)	0.4(C)	0.29(T)	0.08(T)
**7**	Intronic	rs3789679	(+172)C/T [G>A]	na	Na	1:230849694	T (0.09766)	0.1(A)	0.34(A)	0.09(A)
**8**	Exon 2	rs4762*	T174M [ACG > ATG] C3389T	na	Thr>Met	1:230845977	T (0.168)	0.11(T)	0.08(T)	0.04(T)
**9**	Exon 2	rs699*	M235T ; [ATG > ACG] C4072T	na	Met>Thr	1:230845794	T (0.2812)	0.41(C)	0.28(T)	0.08(T)

The table shows the important properties of the studied SNPs and their minor allele frequency (MAF) distributions in our study family, European Caucasian (CEU), Chinese Han (CBH) and Yoruba (YRI) populations. AA, amino acid; Met, methionine; Thr, threonine ,*Significant
